# Trans-Acting Genotypes Associated with mRNA Expression Affect Metabolic and Thermal Tolerance Traits

**DOI:** 10.1093/gbe/evad123

**Published:** 2023-07-01

**Authors:** Melissa K Drown, Marjorie F Oleksiak, Douglas L Crawford

**Affiliations:** Department of Marine Biology and Ecology, Rosenstiel School of Marine, Atmospheric, and Earth Science, University of Miami, Miami, Florida, USA; Department of Marine Biology and Ecology, Rosenstiel School of Marine, Atmospheric, and Earth Science, University of Miami, Miami, Florida, USA; Department of Marine Biology and Ecology, Rosenstiel School of Marine, Atmospheric, and Earth Science, University of Miami, Miami, Florida, USA

**Keywords:** GWAS, eQTL, mRNA expression, WGCNA, metabolism, thermal tolerance

## Abstract

Evolutionary processes driving physiological trait variation depend on the underlying genomic mechanisms. Evolution of these mechanisms depends on the genetic complexity (involving many genes) and how gene expression impacting the traits is converted to phenotype. Yet, genomic mechanisms that impact physiological traits are diverse and context dependent (e.g., vary by environment and tissues), making them difficult to discern. We examine the relationships between genotype, mRNA expression, and physiological traits to discern the genetic complexity and whether the gene expression affecting the physiological traits is primarily cis- or trans-acting. We use low-coverage whole genome sequencing and heart- or brain-specific mRNA expression to identify polymorphisms directly associated with physiological traits and expressed quantitative trait loci (eQTL) indirectly associated with variation in six temperature specific physiological traits (standard metabolic rate, thermal tolerance, and four substrate specific cardiac metabolic rates). Focusing on a select set of mRNAs belonging to co-expression modules that explain up to 82% of temperature specific traits, we identified hundreds of significant eQTL for mRNA whose expression affects physiological traits. Surprisingly, most eQTL (97.4% for heart and 96.7% for brain) were trans-acting. This could be due to higher effect size of trans- versus cis-acting eQTL for mRNAs that are central to co-expression modules. That is, we may have enhanced the identification of trans-acting factors by looking for single nucleotide polymorphisms associated with mRNAs in co-expression modules that broadly influence gene expression patterns. Overall, these data indicate that the genomic mechanism driving physiological variation across environments is driven by trans-acting heart- or brain-specific mRNA expression.

**Significance**

The salt marsh killifish *Fundulus heteroclitus* exhibit large variation in physiological traits assumed to be under stabilizing selection, which should reduce their variation. To discern the heritability of this physiological variation, we took an innovative approach to define the DNA variation that drives mRNA expression linked to physiological variation. This indirect approach revealed many DNA sequence variants associated with physiological variation via their effect on mRNA expression. Surprisingly, these changes were not in the mRNAs themselves, but in unlinked distant genes that are associated with mRNA expression. That is, the vast majority (>95%) were trans-acting. This is surprising because trans-acting effects are found less often than DNA variants within or close to mRNA expression genes. Our results are likely related to the select subset of mRNAs across environments that are linked to physiological variation.

## Introduction

For many complex physiological traits, multiple genetic loci contribute small effects to produce a continuous phenotypic distribution (Gibson 2010; Bernatchez 2016; Boyle et al. 2017). Some traits have been well studied and the polygenic basis established, including human height (Yang et al. 2011; Turchin et al. 2012; Berg and Coop 2014) and egg production in *Drosophila* and chickens (Szydłowski and Szwaczkowski 2001; Jha et al. 2015). Nevertheless, even when complex physiological traits have substantial heritable physiological variation, their genetic basis often is not as well understood (Maher 2008; Zuk et al. 2012; Simons et al. 2018; López-Cortegano and Caballero 2019). For example, metabolism varies by 2- to 3-fold within populations and by orders of magnitude among species (Burton et al. 2011; Pettersen et al. 2018). Some of this variation can be explained by allometric scaling (relationship to body mass) and environment (Burton et al. 2011; Jayasundara et al. 2015; Schulte 2015; Auer et al. 2016; Baris et al. 2016; Pettersen et al. 2018); however, after accounting for these and other covariates, the unexplained heritable variation remains high (Bacigalupe et al. 2004; Rønning et al. 2005; Rønning et al. 2007; Nilsson et al. 2009; Wone et al. 2009). Unexpected and diverse molecular and genetic underpinnings have been identified in other complex traits including thermal tolerance (Healy et al. 2018; Drown et al. 2022), brain size (Zwarts et al. 2015; Hoglund et al. 2020), cardiac cellular ATP production (Baris et al. 2017), and flowering time (Andres and Coupland 2012; Frachon et al. 2017; Grabowski et al. 2017). Thus, the relationships between phenotype and genotype for complex physiological traits are multifaceted and likely to be affected by unfamiliar or unexpected genes (Drown et al. 2022). Moreover, physiological traits are context dependent and often vary in different environments or tissues (Jayasundara and Somero 2013; Baris et al. 2016; Chung et al. 2017; Kellermann et al. 2019; Drown et al. 2021). These attributes make it difficult to predict or identify the genetic variation driving physiological variation. One approach to simplify this multifaceted complexity is to identify the genomic mechanisms affecting mRNA expression that drives phenotypic variation.

mRNA expression variation is often biologically important in that complex or multivariate mRNA expression can explain variation in a diverse suite of traits including thermal tolerance, disease response, and metabolism (Zhang et al. 2019; Huang et al. 2020; Campbell-Staton et al. 2021; Traylor-Knowles et al. 2021; Drown et al. 2022). Some of this expression is physiologically induced; yet mRNA expression is also heritable (Gibson and Weir 2005) and has large variation among common gardened individuals (Oleksiak et al. 2002). Thus, it is possible to identify heritable genetic loci associated with mRNA expression variation. These associations between genetic loci and mRNA expression are identified as expression quantitative trait loci (eQTL), where eQTL mediate the expression of one or many genes. Furthermore, when eQTL controlled mRNA expression affects physiological traits, eQTL may be evolutionary targets for adaptation (Whitehead and Crawford 2006).

**Fig. 1.**
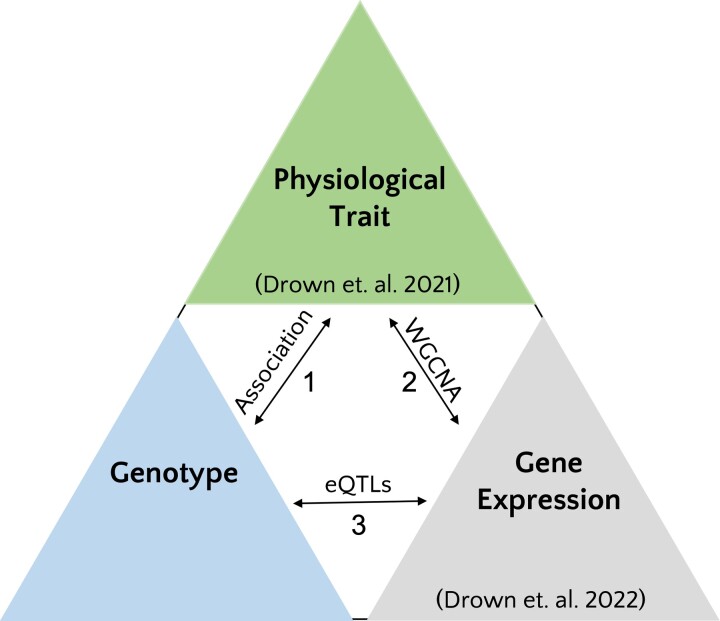
Integrating molecular and genotypic data to understand variation in physiological traits. Physiological trait variation can be driven directly or indirectly (through gene expression) by genotype. To understand the molecular and genetic basis of physiological trait variation, comprehensive data sets can be used to investigate: 1) direct associations between genotype and physiological traits, 2) direct correlations between gene expression and physiological traits, for example, using weighted gene co-expression analysis (WGCNA), and 3) indirect effects of genotype on physiological traits, which may occur when expression quantitative trait loci (eQTL) impact expression of genes underlying physiological traits.

Here, we apply association studies to identify genetic loci directly (genome wide association [GWAS]) and indirectly (eQTL) driving physiological variation. The current study builds upon earlier results where we used three common gardened wild populations to capture natural variation in six complex physiological traits: whole animal metabolism (standard metabolic rate [SMR]), critical thermal maximum (CT_max_), and four substrate specific cardiac metabolic rates (MR_cardiac_) (Drown et al. 2021). Physiological traits and heart and brain mRNA expression were quantified under two ecologically relevant acclimation temperatures (12 °C and 28 °C) in the same individuals. We found little evidence of population divergence in physiological traits (Drown et al. 2021) or differentially expressed mRNAs (Drown et al. 2022). Therefore, we treated all individuals as belonging to a single population and, using a heart- or brain-specific weighted gene co-expression network analysis (WGCNA, Langfelder and Horvath 2008; Healy et al. 2018), found mRNA expression that explained a large proportion of physiological trait variation (Drown et al. 2022). Data from both studies (Drown et al. 2021; Drown et al. 2022) suggest that variation in these physiological traits is driven by both physiological plasticity and heritable genetic variation among individuals. Here, whole genome sequencing was used to identify single nucleotide polymorphisms (SNPs) among the same individuals that were used to quantify physiological and mRNA expression variation allowing us to integrate whole animal (SMR and CT_max_), whole organ (MR_cardiac_), and molecular (mRNA expression) level phenotypes (fig. 1).

Specifically, we address four key questions: 1) are SNPs associated with physiological trait variation (direct drivers), 2) are mRNA expression patterns under genetic control (eQTL), 3) does genetic control of mRNA expression impact physiological trait variation (indirect drivers), and 4) are direct and indirect control mechanisms unique or shared among physiological traits? The expectation is that many SNPs of small effect size explain physiological trait variation for these complex likely polygenic traits. It may be difficult to detect SNPs associated with physiological traits due to small effect size, however, we also expect to find larger effect size cis-acting eQTL that influence mRNA expression patterns previously correlated with physiological traits. Among physiological traits, SNPs and eQTL may be shared as our prior work identified significant trait correlations suggesting a shared genetic and molecular basis. Few studies have integrated data across levels of biological organization in wild populations to address these questions (Morgante et al. 2020), limiting our understanding of genotypic and molecular variation that underlies complex physiological traits. Using this integrative approach, we find that much of the natural variation in complex physiological traits is affected by trans-acting eQTL.

## Results

### Whole Genome Sequencing Results

A total of 172 adult individuals were collected in fall 2018 (F18) from three geographically close (<15 km) populations, and these were individually barcoded and sequenced to an average depth of 4.1× using low-coverage whole genome sequencing (lcWGS). After data processing and filtering (see Materials and Methods), 1,406,282 high-probability variant sites remained (SNPs).

### Population Structure

To determine the genetic structure among populations, we conducted an admixture analysis. Using NGSadmix, we tested seven *K* values where *K* is the number of ancestral populations. We found *K* = 4 to be the best fit based on log-likelihood probability with no clear structure among populations (supplementary fig. S1, Supplementary Material online). The lack of population structure, as well as physiological (Drown et al. 2021) and mRNA analyses (Drown et al. 2022), indicates that there is little demographic structure that could affect mRNA expression, physiological traits, or SNPs.

### Linkage Disequilibrium

To correct for autocorrelation among SNPs contributing to physiological trait and mRNA expression variation, linkage among SNPs was examined using ngsLD (v1.1.0). Similar to prior studies in this species (Dayan et al. 2019; Ehrlich et al. 2020), linkage among sites decayed within 500 bp with an average *R*^2^ below 0.2 within 150 bp and below 0.1 within 300 bp (supplementary fig. S2, Supplementary Material online). Thus, only SNPs associated with the same trait that were >500 bp apart were maintained as independent SNPs not in linkage, and those within 500 bp were pruned to keep the most significant SNP. Few SNPs (5 heart [6.0%], 25 brain [9.3%]) that were significant in the association tests were in linkage, and pruning the SNPs for LD did not substantially change the results.

### Association Studies

We interrogated potential associations between 1,406,282 SNPs and eight phenotypes: six physiological traits: SMR, CT_max_, four substrate specific MR_cardiac_ (glucose, fatty acids, LKA, and endogenous), and two measures of mRNA expression: single mRNA and multivariate mRNA expression. Multivariate mRNA expression used weighted gene co-expression network analysis (WGCNA, Langfelder and Horvath 2008) to identify co-expression mRNAs and group them into modules (MEs). Single mRNA expression was limited to the top ten mRNAs from each WGCNA co-expression module. There were 80 MEs: 39 heart modules and 41 brain modules (supplementary table S1, Supplementary Material online), resulting in 390 single heart mRNAs, and 410 single brain mRNAs (Drown et al. 2022).

#### Direct Genotypic Association to Whole Animal and Whole Organ Level Metabolic and Thermal Tolerance Traits

**Fig. 2.**
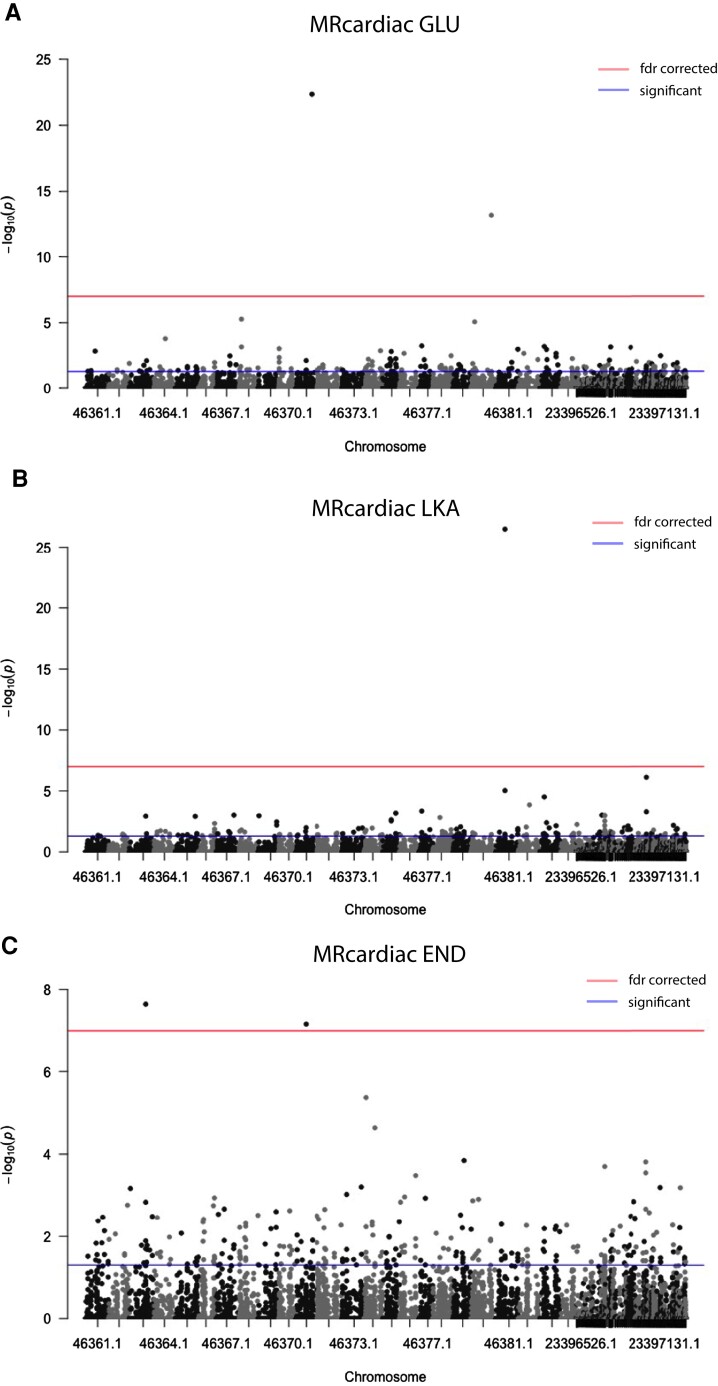
Direct associations: Manhattan plot for SNPs associated with cardiac metabolic rate. Variation in three substrate specific cardiac metabolic rates (substrates: GLU, glucose; LKA, lactate, ketones, and ethanol; and END, endogenous [no substrate added]) was associated with a total of five single nucleotide polymorphisms (SNPs). A) Two SNPs for MR_cardiac_ GLU, B) one SNP for MR_cardiac_ LKA, and C) two SNPs for MR_cardiac_ END. The five SNPs significant after Benjamini–Hochberg *P* value correction are shown above the upper line.

A total of five independent SNPs were significantly associated with three traits (FDR *P* value < 0.05): GLU MR_cardiac_, LKA MR_cardiac_, and END MR_cardiac_ (table 1 and fig. 2). Of these five significant associations, two were associated with GLU MR_cardiac_, one was associated with LKA MR_cardiac_, and the remaining two were associated with END MR_cardiac_ (table 1 and fig. 2). Only one of these SNPs (erfl1, directly associated with END MR_cardiac_) was also a significant eQTL (see below). We did not find significantly associated SNPs for SMR or CT_max_, however, these traits were previously associated with at least one mRNA co-expression module (Drown et al. 2022), described below. The low number of SNPs directly associated with physiological traits is most likely due to the small sample size: Average sample size was 29.60 ± 6.23 (mean ± one standard deviation) individuals per association test (supplementary table S2, Supplementary Material online).

**Table 1:** Single Nucleotide Polymorphisms Significantly Associated with Physiological Traits

Chromosome	SNP location	Trait	FDR *P* value	SNP annotation	Sample size
NC_046380.1	38698336	GLU MR_cardiac_	2.97e^−08^	foxp1b	39
NC_046371.1	29986564	GLU MR_cardiac_	3.15e^−17^	NA	23
NC_046381.1	16773684	LKA MR_cardiac_	4.57e^−21^	kcnb2	31
NC_046363.1	31037486	END MR_cardiac_	3.19e^−02^	erfl1	25
NC_046371.1	19082580	END MR_cardiac_	4.86e^−02^	NA	30

#### Multivariate and Single mRNA Expression

We previously identified heart- or brain-specific mRNA co-expression modules for heart and brain that are associated with variation in the six physiological traits (Drown et al. 2022). Co-expression modules included 39 significant heart modules with 90–554 mRNAs per module and 41 significant brain modules with 142–393 mRNAs per module (Drown et al. 2022). Each module was assigned a module eigengene (ME, the first principal component of multivariate mRNA expression), and each mRNA in the module was assigned a module membership defined as the correlation coefficient between that mRNA and the ME. From each module, we choose the top ten single mRNAs (based on module membership) and used these single mRNA expression values in a series of association tests to identify eQTL. Additionally, we used the ME for each module as a phenotypic value in a separate set of association studies to find SNPs associated with multivariate mRNA expression (eQTL_ME_). This allowed us to identify SNPs that explain mRNA expression patterns previously correlated to acclimation temperature specific physiological traits (SMR, CT_max_, and MR_cardiac_ measured at 12 °C and 28 °C) (Drown et al. 2022). Notably, we did not test all possible mRNAs (∼10,000 mRNAs per hearts or brains); instead, we were interested in discrete relationships between SNPs and specific mRNAs and MEs.

##### Single mRNA Expression Associations

**Fig. 3.**
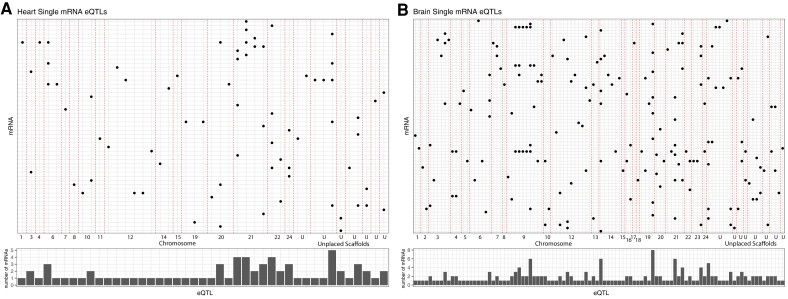
Indirect associations: Expression quantitative trait loci (eQTL) are associated with expression of multiple mRNAs in heart and brain. eQTL for single mRNAs in A) heart and B) brain. Each SNP that is associated with expression of a mRNA (*y* axis) is shown on the *x* axis (sorted by SNP position along each chromosome or scaffold, designed by vertical dashed lines). The bar plots show the number of mRNAs associated with each SNP eQTL (average 1.72 ± 1.01 correlations per SNP for heart [max = 5], average 1.81 ± 1.28 correlations per SNP for brain [max = 8]), sorted by position along each chromosome or scaffold (left to right). All associations are significant with a multiple test corrected *P* value < 0.05 (Benjamini and Hochberg).

For hearts, the 390 single heart mRNAs tested had 79 significant independent genetic associations (FDR *P* value < 0.05) among 56 unique mRNAs with 52 unique SNPs (fig. 3). These 79 significant associations with 56 mRNAs and 52 SNPs occur because a SNP tends to be associated with expression of multiple mRNAs (average 1.58 ± 0.95, max = 5) and an mRNA tends to have more than one eQTL (average 1.48 ± 0.97, max = 4). For brains, the 410 single brain mRNAs tested had 245 significant independent associations among 152 unique mRNAs with 146 unique SNPs (fig. 3). Again, many SNPs were correlated to more than one mRNA (average 1.68 ± 1.08, max = 8), and most mRNAs had more than one significant eQTL (average 2.94 ± 2.49, max = 14). Despite testing a similar number of heart and brain mRNAs, brain had 3.1× more total significant eQTL associations than heart (245 brain vs. 79 heart). There was also a greater proportion of mRNAs with at least one significant eQTL in brain (152/410 [37.10%] compared with heart mRNAs (56/390 [14.36%]) (fig. 3). This difference is not explained by a difference in sample size (i.e., power), which was similar between hearts and brains.

One explanation for a single SNP being associated with multiple mRNAs is that the mRNA affected by the SNP regulates the expression of many genes. This could occur when an eQTL is found in a transcription factor protein or trans-acting regulatory region (e.g., promoter). Additional regulatory regions like LNC-RNA, micro-RNA, or a regulator of DNA methylation or chromatin remodeling could also be important. To determine if an eQTL affected many mRNAs through a transcription factor or other regulatory region, we annotated SNPs and identified those within 5 kb of a transcription factor or other regulatory region (supplementary table S4, Supplementary Material online). Most of the changes were not in transcription factors. Instead, of the seventeen heart eQTL (33.7%) associated with more than one mRNA (hereafter identified as “hotspots”), sixteen were in known protein-coding regions (within an intron or exon) and four of these sixteen (25%) were within an annotated transcription factor (erfl1, tada1, atf1, and zbtb3). The other fourteen were found in protein-coding regions of genes not annotated as transcription factors or other regulatory elements. The one eQTL hotspot not within a known protein-coding region was intergenic and not within 5 kb of an annotated transcription factor. In brains, 63 eQTL (43.2%) were identified as hotspots, and of those, 32 were in known protein-coding regions (within an intron or exon); and only 2 of the 32 (6.25%) were within an annotated transcription factor (erfl1 and zbtb3) whereas 1 intergenic SNP was also within 5 kb of a zinc finger protein (oocyte zinc finger protein XlCOF6-like). One brain eQTL (chd8) was found within a known regulator of chromatin remodeling. Other regulatory regions (LNC-RNA, micro-RNA, other epigenetic regulators) were not identified among the eQTL.

The limited sample size used here (22–35 individuals per association) can cause *P* value inflation. To address this, the qualitative analysis was repeated using a subset of eQTL with FDR *P* value <0.05 and >1.1102e^−16^ (ANGSD documentation as lower bound for reliable likelihood ratio test *P* values). This reduced the number of significant single mRNA associations to 203 (60 heart, 143 brain associations). Examining this subset of significant eQTL the results are qualitatively similar. There are more brain eQTL than heart eQTL and >95% of this subset of significant associations are trans-acting and primarily found in genic regions.

##### Multivariate mRNA Expression Associations

**Fig. 4.**
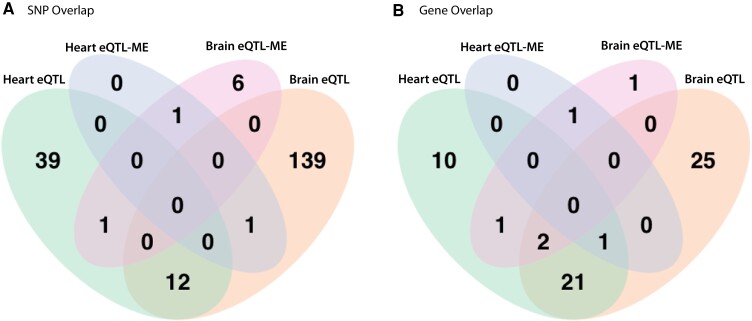
Unique eQTL SNPs and genes are associated with single mRNA and multivariate mRNA co-expression between hearts and brains. A) Overlap among eQTL SNPs associated with single mRNAs and co-expressed mRNA modules (heart and brain modules). B) Overlap among eQTL genes containing SNPs associated with single mRNAs and co-expressed mRNA modules (heart and brain modules).

Among the 39 heart co-expression modules, there were a total of two significant associations (FDR *P* value < 0.05) between two SNPs and two MEs (supplementary table S5, Supplementary Material online). One ME had two significantly associated SNPs and they were found within 50 bp of each other whereas the other SNP was shared for both ME. Among the 41 brain co-expression modules, we found a total of a total of eight significant associations between eight SNPs and seven MEs (fig. 4). MEs are heart- or brain-specific, independent (not correlated to each other), and do not share any mRNAs. One brain ME had two significantly associated SNPs and they were found within 50 bp of each other. Four brain eQTL_ME_ were found within the same gene (erfl1), a known transcription factor.

The eQTL studies for single and multivariate mRNAs indicated significant heritable variation underlying heart- or brain-specific mRNA expression. Previously, multivariate mRNAs were correlated to physiological traits (Drown et al. 2022). Here, we find that the eQTL_ME_ are not associated with modules that are correlated to physiological trait variation. However, the significant eQTL_ME_ indicate that some multivariate expression patterns are heritable they are just not correlated with the traits measured here.

### Trans-acting Effects on mRNA Expression

To better understand the genomic context of eQTL and eQTL_ME_ we determined their proximity to the mRNA(s) they affect. Interestingly, we found that >95% of single mRNA eQTL (97.4% for heart and 96.7% for brain) were trans-acting (defined as SNPs found on a different chromosome or scaffold than that of the mRNA with which they were associated). Specifically, for heart 94.1% (16/17) of eQTL hotspots, and 97.1% (34/35) of eQTL correlated with single mRNAs were trans-acting. For brain 95.2% (60/63) of eQTL hotspots, and 96.4% (80/83) of eQTL correlated with single mRNAs were trans-acting. Although eQTL_ME_ could not be classified as cis- or trans-acting because they affect co-expression modules containing many disparately located mRNAs, we determined whether the eQTL_ME_ were found within 5 kb of mRNAs that were part of the module. All heart and brain eQTL_ME_ were in genes that did not overlap with mRNAs in the associated module. Thus, eQTL_ME_ are not simply cis-acting on a high-ranking mRNA within the module but have broad effects on the expression of many module mRNAs.

### Patterns of Shared Association

For both heart and brain, we looked for eQTL association for ten mRNAs from each co-expression module. Within an ME, the ten mRNAs have some degree of correlation with each other causing them to be grouped into the same module. Thus, we examined whether mRNAs from the same module had more shared eQTL than those from different modules. Surprisingly, within 83% of heart and 67% of brain modules the top ten mRNAs all had unique eQTL. This “uniqueness” is demonstrated in the frequency of shared eQTL within versus among modules: For both hearts and brains, there was fewer shared eQTL within modules than among modules (*t*-test, heart *P* value = 0.027, brain *P* value = 0.008). This eQTL uniqueness would be expected if they were cis-acting, for example, a SNP in the mRNA promoter. Yet, many eQTL are neither near nor within the genes encoding the mRNA they are correlated with. This indicates that these eQTL are trans-acting regulatory SNPs in the sense that they are found outside of the genes that code for modular mRNAs. Similarly, for eQTL_ME_, all were in genes not containing mRNAs from that module. Instead, they were found in different parts of the genome and in genes unassociated with the physiological trait(s) correlated to the module.

Finally, we looked for overlap among SNP sets associated with a given phenotype (physiological traits, heart- or brain-specific single mRNAs, or heart- or brain-specific MEs, fig. 4). First, to address whether the eQTL_ME_ were influencing module expression through action on a single high-ranking mRNA in that module, we assessed whether any SNP was associated with an mRNA and the module containing that same mRNA (overlap in eQTL and eQTL_ME_). That is, if an mRNA belonged to the ME1 heart module, was there a shared SNP or gene containing both an eQTL for the single mRNA and an eQTL_ME_ associated with the ME1 heart module. We found no instances where a SNP or gene contained an eQTL for a single mRNA and an eQTL_ME_ for the module that mRNA belonged to. This suggests that the eQTL_ME_ are not acting on a single mRNAs but represent a more complex mechanism of multivariate mRNA expression control. Second, we examined overlap between hearts and brains for SNPs associated with both single mRNAs and modules. There was only one mRNA (atp7a, 0.13% of total) whose expression was correlated with at least one heart and at least one brain eQTL. However, the eQTL for this mRNA were not the same for heart and brain expression. These data suggest that in hearts versus brains, the variation in atp7a mRNA is affected by different SNPs. There is a limited generality of this finding because we only tested single heart and brain mRNAs that were high-ranking in modules, with only 3% of tested mRNAs shared between heart and brain. Thus, although our results suggest that genetic control of mRNA expression is heart- or brain-specific, examining a larger set of mRNAs expressed in several organs or tissue types would likely be more informative about the role of conserved eQTL among diverse tissue types. An additional caveat is that cell type variation within hearts and brains (e.g., spongy and compact myocardium in heart or neuronal, glial, and endothelial cells in brain) may alter gene expression. Notably, thermal acclimation is known to result in cardiac remodeling in fish, and our prior study (Drown et al. 2021) found evidence of cardiac remodeling between 12 °C and 28 °C acclimated individuals (Gamperl and Farrell 2004; Klaiman et al. 2011; Oellermann et al. 2012; Nyboer and Chapman 2018). Single cell sequencing within hearts and brains could investigate cell type specific gene expression variation that may contribute to patterns described here.

### Genetic Diversity

To compare genetic diversity among SNP sets, heterozygosity at variant sites (H_e_ = 2 ∗ *p* ∗ 1 − *p*; where *p* is the allele frequency) was quantified. Among all 1,406,282 high-probability variant sites, the average heterozygosity was 0.231 ± 0.164 (mean ± standard deviation). Heterozygosity for heart and brain eQTL and brain eQTL_ME_ SNPs was significantly higher than for all SNPs (heart eQTL = 0.328 ± 0.074, brain eQTL = 0.325 ± 0.068, brain eQTL_ME_ = 0.369 ± 0.105). There was no significant difference in H_e_ between brain and heart eQTL and eQTL_ME_ (supplementary fig. S3, Supplementary Material online).

## Discussion

We examined associations among SNPs and specific mRNAs that were previously identified as biologically important based on their membership in co-expression modules. This reduced the number of tested SNPs from ∼1.5 million SNPs across ∼10,000 mRNAs in hearts and in brains to <500 mRNAs in each. Additionally, we use WGCNA (Langfelder and Horvath 2008) to summarize multivariate mRNA expression for co-expressed mRNAs. Using this approach, we summarized ∼10,000 mRNAs in hearts and in brains into 39 heart and 41 brain modules that could be used in our association tests. In comparison to testing each of these mRNAs individually, this may have increased the signal to noise ratio in our mRNA expression data and increased our power by reducing the number of tests (Westra and Franke 2014).

The data suggest that there are many more significant indirect eQTL than direct SNP associations for the physiological traits we examined. Most of these indirect eQTL were trans-acting and in known protein-coding regions (within introns or exons). Many (33.7% of heart and 43.2% of brain) eQTL were hotspots—associated with more than one mRNA. These results are affected by the power of our analyses. Although we used a total of 172 individuals, a minority of individuals (∼25) had heart- and brain-specific and temperature specific mRNA expression data limiting the sample size of the association tests. Although other association studies conducted in wild populations use a similar number of individuals (Hecht et al. 2013; Bourret et al. 2014; Scott et al. 2015; Campbell-Staton et al. 2021), we acknowledge that this may limit our findings in two ways. First, small sample sizes could limit findings to only large effect loci. Yet, the important insights are that nearly all significant eQTL are trans-acting and affect multiple mRNAs that are linked to physiological variation. The observation that nearly all significant eQTL are trans-acting suggests that trans-acting eQTL have larger effect size than cis-acting eQTL, which allowed us to detect them here. An additional explanation could be that more SNPs are in trans than in cis for a given mRNA making it more likely than an eQTL also is trans. This is possible as there is no significant difference between the detected proportion of trans eQTL from that expected based on SNP distribution among chromosomes (chi-square test *P* > 0.05). Second, small sample sizes may increase the risk of detecting spurious associations. Yet, the observation that eQTL were trans-acting and because they affected multiple mRNAs indicates that the eQTL are not spurious. That is, it is unlikely that multiple independent associations would spuriously be significant for a given eQTL. Thus, although all our conclusions are based on the limits of detection, these limits indicate the importance of trans-acting factors associated with physiologically important mRNA expression. Still, we caution against making assumptions about the role of specific SNPs that have been identified here in explaining physiological traits or gene expression in other populations. This is both due to the limited sample size and the lack of direct evidence (e.g., a traditional quantitative trait loci [QTL] study) for potential quantitative trait loci and eQTL. The alternative hypothesis is that these loci are not causative. Yet, using a subset of significant eQTL to obtain more conservative estimates, the overall conclusion of mostly trans-acting eQTL is still found. An alternative approach would be breeding individuals with low and high gene expression values and examining the heritability of gene expression patterns in relation to a specific SNP. Similarly, specific gene expression could be interrogated using qPCR of a common gardened individual with known trait measurements. Instead, we focus the discussion on general patterns that speak to the genetic and molecular control of physiological traits.

## Direct Genetic Control of Physiological Traits

Three out of six physiological traits (GLU, LKA, and END MR_cardiac_) were associated with five SNPs (table 1 and fig. 1). All three of these traits are for cardiac metabolism, potentially reflecting less complex genomic architecture than standard whole animal metabolism or CT_max_. Three of the five SNPs were within annotated genes: foxp1b (GLU MR_cardiac_), kcnb2 (LKA MR_cardiac_), and erfl1 (END MR_cardiac_). Foxp1b is forkhead box protein and erfl1 is a repressor factor; both are involved in negative regulation of transcription by RNA polymerase II (Cheng et al. 2007; Liu and Patient 2008). Kcnb2 is a voltage gated potassium channel, with various functions including but not limited to regulation of neurotransmitter release, heart rate, insulin release, and smooth muscle contraction (Hristov et al. 2012; Li et al. 2022). These three genes are not found in any of the heart or brain mRNA co-expression modules. Yet, one direct SNP (erfl1) is associated with END MR_cardiac_ and contains different SNPs that are eQTL associated with mRNA expression in hearts and brains and three brain eQTL_ME_. These erfl1 SNPs are all linked (within 500 bp of each other).

Our study did not find any direct association with *F. heteroclitus*’ CT_max_, but a prior study found 47 candidate SNPs associated (Healy et al. 2018). Similarly, none of our SNPs were directly associated with standard metabolic rate although prior studies have found multiple candidate QTL associated with metabolic rate (Jacobson et al. 2006; Palomar et al. 2019). It is likely that this study missed these because of the complex nature of metabolic and thermal tolerance traits with many small effect loci rather than one or few large effect loci explaining these traits (Wentzell et al. 2007; Burton et al. 2011; Csilléry et al. 2018; Healy et al. 2018). Multiple studies provide evidence for a polygenic basis of both traits (Healy et al. 2018; Barghi et al. 2019). Our approach, which is best suited to detect large effect associations, is underpowered. Thus, although we detected few direct drivers of physiological trait variation, this is unlikely to be representative of the biology. Using a multivariate approach where genotypes at many SNPs could be used to explain these traits may improve our detection of associated SNPs, as has been done in other studies (Bourret et al. 2014; Healy et al. 2018; Barghi et al. 2019). Instead of this multivariate approach, we focus on mRNA expression patterns that may indirectly impact physiological traits. The ability of this approach to identify indirect drivers (eQTL for mRNAs linked to physiological traits) may be due to high effect sizes of eQTL compared with QTL, especially for multivariate mRNA expression (Westra and Franke 2014; Boyle et al. 2017). That is, whereas physiological traits are complex and affected by 100 s to 1,000 s of loci, mRNA expression is likely to be affected by fewer loci; thus, each eQTL will have a larger effect because fewer polymorphisms are involved (Boyle et al. 2017).

## Are mRNA Expression Patterns under Genetic Control?

We found eQTL for 14.6% and 20.7% of the tested heart and brain mRNAs, respectively, suggesting that genetic variation is important in explaining these mRNA expression patterns (fig. 2). Interestingly, the majority (97.4% for heart and 96.7% for brain) of eQTL that we detected were found on different chromosomes than the associated mRNA (trans-acting). The characterization of trans-acting eQTL was not due to the presence of many small scaffolds with 78.4% of the genome found on 24 large (>28 Mb) chromosomes (https://www.ncbi.nlm.nih.gov/datasets/genome/GCF_011125445.2) and an expected proportion of eQTL found on scaffolds (fig. 3; 25.3% of heart eQTL and 15.9% of brain eQTL). The higher number of trans- versus cis-acting eQTL could reflect a higher effect size of trans- versus cis-acting eQTL for the selected mRNAs expression or may be biased by our detection method as discussed below. The relative role of cis- and trans-acting factors is often examined; however, many studies have found a prominent role of cis elements in comparison to trans elements (Kitano et al. 2019, although see Hoglund et al. 2020). Here, we find the opposite with few (<5%) of all eQTL in heart and brain being cis-acting (found on the same chromosome). If trans-acting factors have a larger effect size, this may be explained by our increased likelihood of detecting trans versus cis factors; however, other studies often struggle with the detection of trans-acting factors, which is often attributed to their lower effect size (Nica and Dermitzakis 2013; Westra and Franke 2014). Thus, our ability to detect trans-acting factors is unique and may be explained by the selection of specific mRNAs that are central to co-expression modules for our tests. That is, we may have enhanced our ability to identify trans-acting factors by looking for SNPs associated with mRNAs in co-expression modules that are known to be correlated with the expression of 10 s or 100 s of other genes. Thus, by examining a select set of single mRNAs, we may have captured many transcription factors or other regulatory elements likely to have widespread effects on broad gene expression patterns. As eQTL effects are often context dependent, our examination of heart- and brain-specific and temperature specific gene expression also may have contributed to the number of significant trans-acting elements we have detected (Westra and Franke 2014; Boyle et al. 2017). In contrast, other studies may use the whole transcriptome (rather than specific organs or tissues) and rarely examine mRNA expression under multiple environments. The frequency of trans-acting effects is important because it is often undetected and unappreciated. (Price et al. 2011). Metzger et al. suggested that trans regulatory mutations may be more common but do not persist over evolutionary time (between species) when compared with cis-acting factors. Our results echo this finding in that we emphasize the importance of trans-acting factors for within species gene expression. Whether the trans-acting factors persist due to neutral or are affected by natural selection is unclear. However, 44.2% of heart and 11.2% of brain trans-acting SNPs defined here are associated with mRNA expression that affect important physiological processes. Thus, either these trans SNP are non-neutral or the mRNA and the traits they appear to affect are evolutionary neutral.

Whereas we did not classify SNPs associated with MEs as cis or trans because there is no single genomic location for a module, we did examine whether SNPs associated with modules were found in genes that produced mRNAs that were part of that module. We found no association between SNP identity and module membership. That is, nearly all the eQTL for MEs were not found in or within 5,000 bp of the genes for the mRNAs in our MEs. Although 61.3% of eQTL and 80% of eQTL_ME_ were within genic regions (introns or exons), few eQTL (7.5%) were found within 5 kb of transcription factors or other regulatory elements. The percentage of eQTL found in or near transcription factors is similar to the percentage of transcription factors found globally in eukaryotic genomes and indicates that there is not an enrichment for eQTL or eQTL_ME_ within or near transcription factors (Riechmann 2002). The same is true of brain eQTL hotspots (6.25% within or near transcription factors), however, 25% of heart eQTL hotspots were within an annotated transcription factor. This is 2- to 5-fold enriched when considering the proportion of protein-coding genes that are transcriptional regulators among organisms: ∼8% (1,600/20,000) in humans (Lambert et al. 2018), 12.6% in zebrafish (3,302/26,602) (Armant et al. 2013), and 4–5% in yeast (264/6,000) (de Boer and Hughes 2012). Similarly, the large number of eQTL and eQTL_ME_ (61.3–80%) found within genic regions (introns or exons) exceeds expectations (∼2% of eukaryotic genomes are protein coding). These data suggest that trans-acting factors may include more than just the annotated transcription factors or that annotations are lacking. Prior studies have reported an enrichment within and near genic regions for trait associated SNPs (Li et al. 2012; Watanabe et al. 2019) and a high likelihood of trait associated SNPs being eQTL (Nicolae et al. 2010). Our data have similar findings with an enrichment of eQTL and eQTL_ME_ within genic regions.

SNPs in three genes had both eQTL_ME_ and eQTL for single mRNAs (erfl1, LOC105931894, and pnpla7a). Of these, one gene, erfl1, is a known transcription factor and had four intronic eQTL_ME_ and contained five SNPs associated with 13 single mRNAs. The SNPs associated with single mRNAs were not the same as those associated with ME expression, suggesting that eQTL_ME_ were functionally independent from any single mRNA. This demonstrates that ME expression is not driven by the effect of an eQTL on a single high-ranking mRNA within the module.

The approximately 25 individuals for any one SNP limit the conclusion on the diversity of mRNAs and molecular mechanisms affecting the six physiological traits. Yet, we find many significant eQTL for both single and multivariate mRNA expression where a vast majority were trans-acting or distant to the variable mRNA loci. These findings suggest that our approach may allow for better detection of trans-acting elements, which may affect expression of dozens or hundreds of co-expressed mRNAs.

Finally, we find that eQTL and brain eQTL_ME_ have greater heterozygosity (frequency of variant alleles) when compared with all 1,406,282 variant sites used in this study. Heart eQTL_ME_ approached significance (*P* = 0.056) but was not different from all SNPs likely due to there being only two SNPs in this subset. This may be explained by the association approach as less variable sites have less variance among individuals that can be used to explain variance in the physiological traits and mRNA expression patterns. It is also possible that allele frequency differences are biologically relevant. If eQTL and eQTL_ME_ sites are under directional selection, we might expect a loss of genetic diversity in these sites. Yet, we find that heterozygosity (frequency of variant alleles) is higher in eQTL and eQTL_ME_ when compared with all SNPs. Higher heterozygosity may be due to genetic redundancy, as indicated by the presence of different SNPs underlying eQTL and eQTL_ME_ and the association of correlated physiological traits with different underlying loci. These patterns could be driven by spatial or temporal variation in selection preventing allelic fixation and aiding in the maintenance of biologically important variation at the SNP and mRNA level.

## Are Genetically Controlled mRNA Expression Patterns Likely to Impact Physiological Traits?

Heritable genetic variation impacts mRNA expression (Gibson and Weir 2005), a molecular level phenotype that is well established as important in physiological response to the environment (McCairns and Bernatchez 2010; Albert and Kruglyak 2015; Dayan et al. 2015; Zhang et al. 2019; Campbell-Staton et al. 2021). Thus, our finding of genetic links to mRNA is not surprising, even with a small sample size. What is unique in our data is the ability to examine relationships among genotype and gene expression (eQTL) of individuals from wild populations and interpret them in the context of known correlations between gene expression and physiological traits. This may be especially important for improving our ability to detect biologically important genetic variation (Albert and Kruglyak 2015) because gene expression is a relatively simpler trait that may be controlled by fewer, larger effect, and easier to detect loci (Boyle et al. 2017).

Previously, we found that up to 82% of the variation in several temperature specific (12 °C or 28 °C) metabolic and thermal tolerance traits could be explained by co-expression among hundreds of mRNAs grouped into modules (Drown et al. 2022). However, we could not parse the roles of plasticity versus heritable mRNA expression variation. Here, we show that expression of single mRNAs found within these modules and multivariate module expression is associated with genetic variation. This suggests that a significant portion of several physiological traits may be explained by heritable mRNA expression variation. Specifically, genetic variation in heart mRNA expression is linked to 12 °C FA MR_cardiac_, 12 °C SMR, and 12 °C LKA MR_cardiac_. Heritable variation in brain mRNA expression is linked to 28 °C CT_max_ (supplementary table S6, Supplementary Material online). This provides further evidence that mRNA expression patterns impacting these physiological traits are under genetic control and heritable.

Prior studies have demonstrated that heritable mRNA expression variation can impact diverse physiological traits. For example, behavioral maturation from hive worker to forager between honey bee subspecies is partially attributed (up to 30%) to heritable variation in brain gene expression (Whitfield et al. 2006). Various organisms including sea turtles (Tedeschi et al. 2016), maize (Frova and Gorla 1993), fruit flies (Sejerkilde et al. 2003), and fish (Fangue et al. 2006; Heredia-Middleton et al. 2008) among others exhibit heritability of heat shock protein expression, allowing them to respond to environmental temperature variation. Here, we found that biologically important single and multivariate mRNA expression related to physiological traits has a genetic basis and is heritable. This is similar to studies that have found overlap among QTL and eQTL sets (Wentzell et al. 2007; Carrasco-Valenzuela et al. 2019). Yet, the finding that a single eQTL is significantly associated with 100 s of co-expressed mRNAs is unique. Further, many of the modules were associated with more than one eQTL, suggesting that there is substantial genetic variation contributing to gene expression patterns related to physiological traits. The allelic variation in gene expression provides the raw material for evolution and may explain the vast interindividual variation in physiological traits that we have measured (Drown et al. 2021; Drown et al. 2022).

## Are Physiological Traits Genetically Independent?

Here, we have shown that mRNA expression patterns previously correlated with physiological traits are associated with a suite of mostly trans-acting eQTL, suggesting genetic control. One additional potential outcome of this study was to determine if physiological traits had a shared genetic basis. This was an important avenue to explore as our prior studies found correlation among traits (Drown et al. 2021) and shared patterns of mRNA expression among correlated traits (Drown et al. 2022). For example, we previously found that 12 °C FA MR_cardiac_ was positively associated with 12 °C SMR and that these traits were both associated with two MEs (heart ME4 and heart ME5). If these traits also had a shared genetic basis, we expected eQTL for heart ME4 and heart ME5 to be shared. No eQTL_ME_ were associated with multivariate expression of trait associated modules; however, 35 eQTL were associated with expression of single genes belonging to trait associated modules. There were five shared SNPs among eQTL within or associated with genes in trait associated modules. Of these SNPs, three were shared between modules associated with a shared trait (supplementary table S6, Supplementary Material online). Specifically, heart ME3 and heart ME5, both associated with 12 °C FA MR_cardiac_, share a SNP (NW023397088_1791840), and heart ME4 and heart ME9, both associated with 12 °C SMR, share a SNP (NC046366.17066247). Notably, three other SNPs were shared among modules correlated to different traits (one SNP shared between modules associated with 12 °C SMR and FA MR_cardiac_, two SNPs shared between modules associated with 12 °C SMR and 12 °C LKA MR_cardiac_). This provides evidence that there may be a shared genetic basis or genetic control of these metabolic traits. Notably, this was only true of heart modules with no shared SNPs among the three brain modules, which were all associated with 28 °C CT_max_. Additionally, the five SNPs associated directly with physiological traits were not shared among traits and do not overlap with any eQTL. One known transcription factor, erfl1, contains multiple SNPs that are either directly associated with a trait (one SNP, 12 °C END MR_cardiac_), are eQTL (one heart, five brain), or are heart or brain eQTL_ME_ (four SNPs).

Overall, the limited overlap among SNPs associated directly or indirectly (eQTL) with physiological traits within hearts or brains was surprising. This may be due to our limited ability to detect small effect QTL, and we might expect greater overlap between QTL and eQTL if more mRNAs were tested across more individuals. However, within the power of our data, we detected diverse and complex molecular mechanisms correlated with physiological trait variation. Heart- or brain-specific expression patterns appear to be under unique genetic control, and multivariate mRNA expression is not explained by a single eQTL impacting mRNA expression of a gene highly correlation to a given module.

Thus, although different traits are correlated to the same ME, the nucleotide polymorphism, or genetic control, of mRNA expression is distinct. This suggests that there is substantial genetic variation underlying the physiological traits we have measured, with a diversity of molecular and genetic mechanisms contributing to trait variation. The paradoxical genetic independence of physiologically related traits (here metabolism and thermal tolerance) is not uncommon (see Van Herrewege and David 1980; Baker et al. 2015; Healy et al. 2018) and emphasizes that these traits may still be evolutionarily distinct, although they are linked at the molecular or physiological level.

## Conclusions

Relationships among genotype, gene expression, and physiological traits explain biologically important natural variation found in wild populations. In particular, substantial and diverse genetic variation impacts these traits through direct and indirect (eQTL and eQTL_ME_) mechanisms. Demonstrated here, much of the mRNA expression variation is associated with a diverse set of trans-acting eQTL. Surprisingly, these trans-acting eQTL are unique even for mRNAs that affect multiple traits. Under a simpler genetic architecture, we may expect mRNAs that have a shared association with cardiac and whole animal metabolism to also share the same trans-acting eQTL, but this does not occur. Instead, the mRNA expression changes that affect multiple physiological traits are associated with different trans-acting SNPs. Finally, the SNPs directly or indirectly associated with physiological traits have greater heterozygosity (genetic variation) compared with all SNPs, and this greater genetic variation likely contributes to *F. heteroclitus*’ well characterized resilience and plasticity (reviewed in Burnett et al. 2007; Crawford et al. 2020) in the face of novel environments. Additional studies are needed to demonstrate causative relationships between SNPs, mRNAs, and traits. Yet, there are known functional relationships between SNP variance and mRNA expression, and these patterns are heritable. It is possible that the eQTL identified here are linked to causal variants and are not the causal variants per se, however, we do demonstrate in finding SNPs and eQTL associated with physiological trait variation that these traits are under genetic control at least partially driven by heritable mRNA expression patterns. Together, our data suggest genetic control of biologically effective, mRNA expression (expression that impacts physiological traits), which in turn, may impact fitness.

## Materials and Methods

1. Sample collection: Fin clips were taken from adult *F. heteroclitus* collected along the central coast of New Jersey, United States near the Oyster Creek Nuclear Generating Station (OCNGS), which produces a thermal effluent that locally heats the water. Three populations were sampled: 1) north reference (N.Ref; 39°52′28.000N, 74°08′19.000W), 2) south reference (S.Ref; 39°47′04.000N, 74°11′07.000W), and 3) a central site located between the southern and northern references that is within the OCNGS thermal effluent (TE; 39°48′33.000N, 74°10′51.000W). The TE population used here differs by 4 °C in habitat temperature from the two reference populations (average summer high tide temperature 28 °C N.Ref and S.Ref, and 32 °C for TE) but is otherwise ecologically similar (Drown et al. 2021) (Dayan et al. 2019). Fin clips were collected in fall 2015 (F15), fall 2018 (F18), spring 2019 (S19), fall 2019 (F19), and fall 2020 (F20) and stored in GuHCl buffer. DNA was extracted using carboxyl coated magnetic beads. The DNA quality was assayed using gel electrophoresis and spectrophotometry to ensure high molecular weight and low contamination.
2. Library preparation: The analysis presented here uses a subset of samples that were part of a larger sequencing run. A total of 1,121 individuals were sequenced (supplementary table S3, Supplementary Material online). All samples were quantified in triplicate using spectrophotometry and normalized to 100 ng for sequencing library preparation. The whole genome sequencing library was prepared using a tagmentation approach. Briefly, DNA was digested with an in-house purified Tn5 transposase (as in Picelli et al. 2014) loaded with partial adapter sequences. After tagmentation, the fragmented DNA was amplified using barcoded primers such that each individual sample would contain a unique i7 and a plate level (1 per 96 samples) i5 barcode. This allowed for unique dual indexing of up to 768 individuals. After barcoding, samples were combined into two pools (560 samples each) and each pool amplified and then sequenced on a single lane of Illumina HiSeq 3000. These single sequencing lanes were assessed to determine coverage balance among samples, and the same libraries were sequenced across an additional four lanes each. For all sequencing runs, a greater relative amount of library for F18 samples was added to the pool to achieve higher coverage because whole animal, whole organ, and molecular (mRNA expression) level phenotypic data were available for these individuals.
3. Raw sequence analysis: We followed best practices for lcWGS data processing as in Lou et al. (2021). Briefly, adapter sequences and low-quality bases were trimmed using Trimmomatic (v0.39) (Bolger et al. 2014). Flash (v1.2.11) (Magoč and Salzberg 2011) was used to combine overlapping reads and to parse singletons and paired reads. Singletons and paired reads were mapped separately using BWA mem (v0.7.17) and resulting sam files converted to bam files using samtools (v1.3.1) (Danecek et al. 2021). The first and second sequencing runs were processed separately until BAM files were produced and found to be of similar quality assessed by comparing total percentage of mapped reads and levels of dually mapped reads before combining for the remaining file processing steps. Picard (v2.26.4) was used to add read group information, which is needed for duplicate marking downstream. After combining all mapped reads for a single individual, BAM files were further filtered for mapping quality using samtools and overlapping reads softclipped using bamutil (v1.0.15). Finally, Picard (v2.26.4) was used to mark duplicate reads.
4. Variant calling: Two variant calling pipelines were used. First, Freebayes (v1.0.2) was used to call variants, and the resulting VCF file was filtered using VCFtools (v0.1.16). VCFtools filters were to include only biallelic sites, >5% minor allele frequency, <5% missingness per individual, and <10% missingness per site. This resulted in 1,406,282 high-probability variant sites. ANGSD (v0.935) (Korneliussen et al. 2014), which is designed for use with lcWGS data, was then used to obtain a genotype likelihood beagle file containing the previously identified high-probability variant sites from Freebayes and VCFtools. This approach is similar to other studies using lcWGS data where variant calling may be sensitive to specific tool use.

### Phenotypic Data

Methods and analyses for phenotypic data are described in previous publications (DeLiberto et al. 2020; Drown et al. 2020; Drown et al. 2021; Drown et al. 2022). Briefly, all phenotypes were measured after common gardening and under two temperature acclimation conditions. Whole animal phenotypes included temperature specific whole animal metabolism (standard metabolic rate [SMR]) and critical thermal maximum (CT_max_, a measure of thermal tolerance) measured at and after acclimation to 12 and 28 °C. Heart-specific phenotypes included four substrate specific cardiac metabolic rates (MR_cardiac_, substrates: glucose [GLU], fatty acids [FA], lactate + ketones + ethanol [LKA], and endogenous [END, no substrate added]) measured for half the individuals at 12 °C and half at 28 °C.

Hearts and brains were collected after measuring MR_cardiac_ and stored in chaotropic buffer for mRNA expression analysis. The mRNA data include heart- or brain-specific expression counts for single mRNAs and heart- or brain-specific module mRNA expression (ME) from a whole genome co-expression network analysis (WGCNA, v1.70–3, Langfelder and Horvath 2008; Drown et al. 2022). The WGCNA approach summarizes mRNAs with correlated expression into co-expression modules, calculates a principal component of module expression for each individual (ME), and assigns a rank to single mRNAs within the module (module membership) based on their correlation to the ME. Here, we use the first principal component of module expression (ME) as a multivariate molecular level phenotype that may be predicted using genotype likelihoods. In addition, we examined association between genotype likelihoods and the top ten mRNAs for each module (based on module membership).

1. Association studies: All results are reported from the score test conducted in ANGSD using -doAsso 2 with default filters (-minHigh 10, -minCount 10). The sample size for each association can be found in supplementary table S2, Supplementary Material online and is limited by the availability of phenotypic and mRNA expression data (a subset of the 172 genotyped individuals). For all phenotypes, acclimation temperature was included as a covariate. For SMR, CT_max_, and MR_cardiac_, acclimation order (individuals were acclimated to 12 °C then 28 °C or 28 °C then 12 °C) was included as an additional covariate. For SNP associations to mRNA expression, heart and brain mRNA expression were examined as separate phenotypes. *P* values for genotype to phenotype associations were corrected for multiple testing using the Benjamini–Hochberg approach (Benjamini and Hochberg 1995) and significant associations identified as those with a corrected *P* value < 0.05. To examine patterns among independent SNPs, in cases where SNPs associated with the same phenotype were within 500 bp of each other, we pruned SNPs to keep the most significant SNP for each association and removed any within 500 bp of that SNP.
2. Annotation of significant SNPs: A bed file was generated from a SNP list using the genomic region for the SNP as the SNP location—1 bp: SNP location. Bedtools intersect was used to obtain annotation information from the .GTF file for the current *F. heteroclitus* genome.
3. Statistical analysis: Data visualization and statistical analyses were conducted in R (v 4.0.5). An annotated script is available on Github (https://github.com/mxd1288/Genotypic_drivers.git). Association tests were carried out using ANGSD, as described above, and the likelihood ratio test values used to calculate *P* values for each SNP to trait association calculating using a one-sided noncentral chi-squared distribution (pchisq in R). *P* values were corrected within each set of trait associations using p.adjust in R with the Benjamini and Hochberg method (Benjamini and Hochberg 1995).

## Supplementary Material

evad123_Supplementary_Data

## Data Availability

All whole genome sequences are available in the NCBI SRA under PRJNA967634, PRJNA967633, PRJNA967630, and PRJNA967626. RNA expression data are available on NCBI under PRJNA796010. Metadata are available in Dryad repository: doi:10.5061/dryad.mw6m9061g.
