# Supplementary material for: Trans-Acting Genotypes Associated with mRNA Expression Affect Metabolic and Thermal Tolerance Traits
Source: Genome Biol Evol. 2023 Jul 1;15(7):evad123. doi: 10.1093/gbe/evad123 (PMC10370451; doi:10.1093/gbe/evad123)
Supplement: evad123_Supplementary_Data [file evad123_supplementary_data.zip › Drown_Trans-acting_Drivers_SupplementalFigures_GBE.docx]

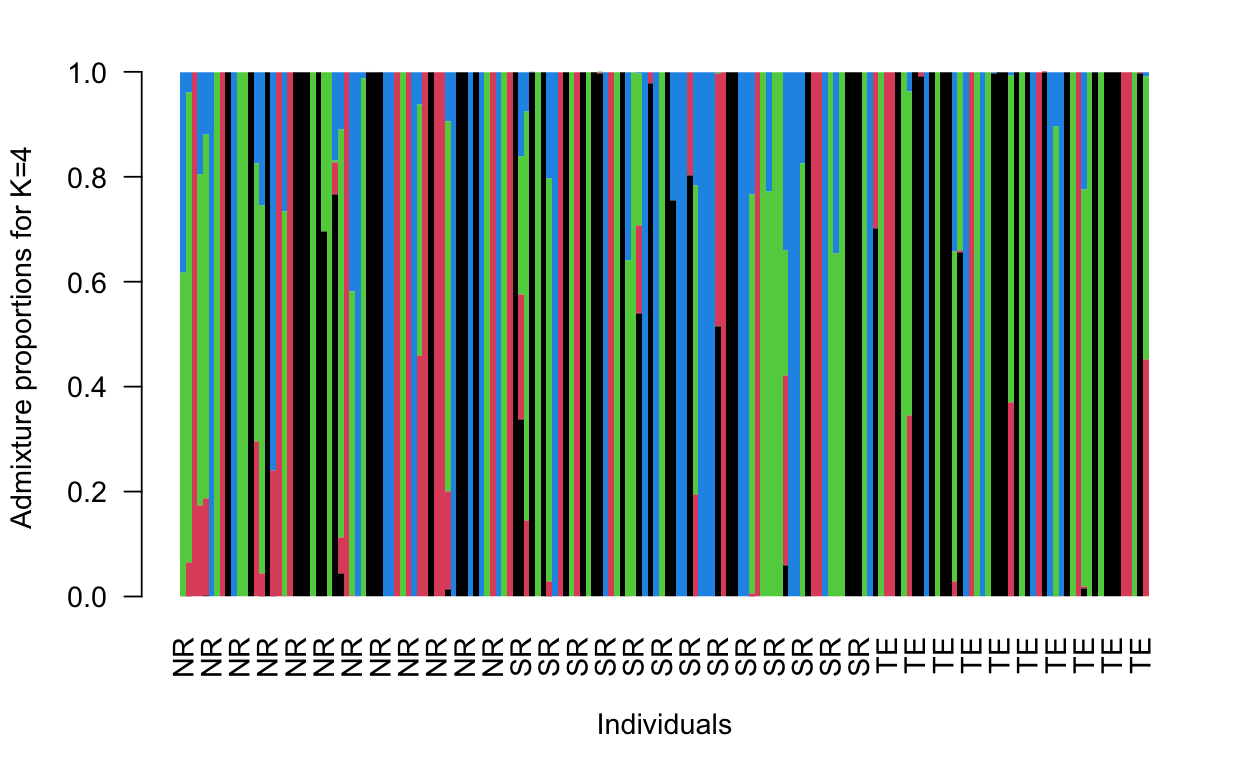


**Figure S1: No evidence in admixture analysis of population structure among samples.** The best K=4 based on log-likelihood probability for 172 individuals collected from three population in fall 2018: North Reference (NR), South Reference (SR), and Thermal Effluent (TE) populations.

**
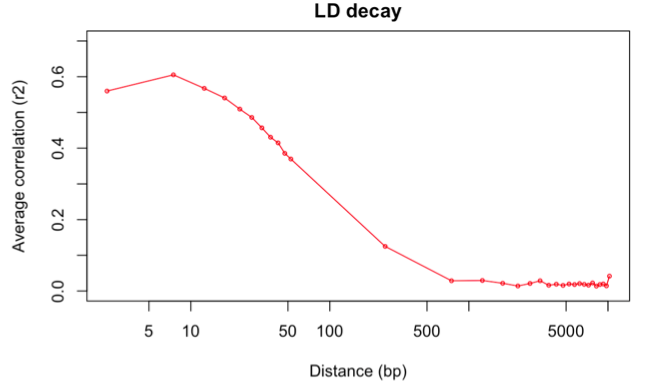
**

**Figure S2: Decay of linkage disequilibrium.** Linkage among SNPs decays to an average correlation (y-axis) of <0.1 within 300bp.

**Figure S3: Heterozygosity among all single nucleotide polymorphisms and eQTL sets.** Heterozygosity was significantly higher for SNPs identified as eQTL_ME_ and eQTL (p<0.05) when compared to all SNPs except for heart eQTL_ME_ (p=0.06).
