# Supplementary material for: Trans-Acting Genotypes Associated with mRNA Expression Affect Metabolic and Thermal Tolerance Traits
Source: Genome Biol Evol. 2023 Jul 1;15(7):evad123. doi: 10.1093/gbe/evad123 (PMC10370451; doi:10.1093/gbe/evad123)
Supplement: evad123_Supplementary_Data [file evad123_supplementary_data.zip › Drown_GBE_ReviewerResponses2.docx]

line 291-- I'm afraid I was unclear in my comments here--  "To compare genetic diversity among SNP sets"
Genetic diversity measures normally take into account invariant sites-- i.e.,  a gene with 100 SNPs and a gene with 1 SNP will have different genetic diversities (and heterozygosities, as the invariant sites will be homozygous factoring them in reduces heterozygosity).
Here, you've  looked at heterozygosity at already variant sites-- a gene with 100 SNPs and a gene with 1 SNP could, in principle, have the same amount of heterozygosity with this measure. You're therefore comparing the frequencies of variant alleles, really, not heterozygosity.  I'm persisting with this point as I know at least one reviewer was confused by this issue (and because clarity is important).
The discussion on lines 454-460 still holds, if you simply replace 'heterozygosity' with 'allele frequencies'. Please also insert 'heterozygosity at _variant_ sites' somewhere in the vicinity of Line 291.

The recommended changes have been made for clarity.

Line 291: “To compare genetic diversity among SNP sets, heterozygosity at variant sites (H_e_ = 2*p*1-p; where p is the allele frequency) was quantified.”

Line 450-460: “Finally, we find that eQTL and brain eQTL_ME_ have greater heterozygosity (frequency of variant alleles) when compared to all 1,406,282 variant sites used in this study. Heart eQTL_ME_ approached significance (p=0.056) but was not different from all SNPs likely due to there being only two SNPs in this subset. This may be explained by the association approach as less variable sites have less variance among individuals that can be used to explain variance in the physiological traits and mRNA expression patterns. It is also possible that allele frequency differences are biologically relevant. If eQTL and eQTL_ME_ sites are under directional selection, we might expect a loss of genetic diversity in these sites. Yet, we find that heterozygosity (frequency of variant alleles) is higher in eQTL and eQTL_ME_ when compared to all SNPs. Higher heterozygosity may be due to genetic redundancy, as indicated by the presence of different SNPs underlying eQTL and eQTL_ME_ and the association of correlated physiological traits with different underlying loci.”

line 405-- don't forget to remove the quotes

Thank you for catching this error, it has been corrected.

line 18, 30, 89, 148, 163, etc. 'tissue-specific' is used to describe heart or brain gene expression, when what you've done is something different-- organ-specific gene expression.  I apologise if this seems pedantic, but from the abstract I was expecting single-cell data (though would have been surprised in reality, as that is would be a hugely expensive study!).
'Organ-specific' is unwieldly, but consider replacing tissue-specific with 'heart- or brain-specific'. Alternatively, as a compromise, introduce the data by explaining by explaining that 'tissue-specific' refers to cardiac and brain tissue, in reality a mix of cell and tissue types, and then explain that you'll refer to this as 'tissue specific' expression anyway.

Tissue specific has been replaced with heart or brain specific where appropriate including changes on lines 18, 30, 89, 224, 229, 264, 280, 313, 401, 532, 625, 628, and 629.

The use of “tissue” has also been changed in several places

- Fig. 3 and Fig. 4 legend (now “hearts and brains” instead of “heart and brain tissues”)
- line 97 (“..allowing us to integrate whole animal, tissue..” now reads “…allowing us to integrate whole animal, whole organ..”
- Line 148: replaced “tissue level” with “whole organ level”
- Line 176: now “(~10,000 mRNAs per hearts or brains)”
- Line 192: “This difference is not explained by a difference in sample size (i.e., power), which was similar between hearts and brains.”
- Line 274: “Second, we examined overlap between hearts and brains for SNPs associated with both single mRNAs and modules.”
- Line 277: “These data suggest that in hearts *vsersus* brains the variation in atp7a mRNA is affected by different SNPs.”
- Line 280-282: “Thus, while our results suggest that genetic control of mRNA expression is heart- or brain-specific, examining a larger set of mRNAs expressed in several organs or tissue types would likely be more informative about the role of conserved eQTL among diverse tissue types.”
- Line 301: “This reduced the number of tested SNPs from ~1.5 million SNPs across ~10,000 mRNAs in hearts and in brains to <500 mRNAs in each.”
- Line 303: “Using this approach, we summarized ~10,000 mRNAs in hearts and in brains into 39 heart and 41 brain modules that could be used in our association tests.”
- Line 404: “In contrast, other studies may use the whole transcriptome (rather than specific organs or tissues) and rarely examine mRNA expression under multiple environments.”
- Line 529: “Overall, the limited overlap among SNPs associated directly or indirectly (eQTL) with physiological traits within hearts or brains was surprising.”
- Line 593: “For all sequencing runs, a greater relative amount of library for F18 samples was added to the pool to achieve higher coverage because whole animal, whole organ, and molecular (mRNA expression) level phenotypic data was available for these individuals.”
- Line 626: “Hearts and brains were collected…”
